# From dangerous branches to urban banyan: Facilitating aerial root growth of *Ficus rubiginosa*

**DOI:** 10.1371/journal.pone.0226845

**Published:** 2019-12-30

**Authors:** Angela T. Moles, Ashika Jagdish, Yameng Wu, Suzanna Gooley, Rhiannon L. Dalrymple, Phoebe Feng, Jennifer Auld, Georgia Badgery, Matilda Balding, Andrew Bell, Nora Campbell, Mark Clark, Michelle Clark, Kyle M. Crawford, Oliver de Lorenzo, Amelia Fletcher, Zoe Ford, Haley Fort, Simon B. Z. Gorta, Alexander Hagan, Frank A. Hemmings, Gabriella S. Hoban, Thomasine Hulme, Kit King, Anish Kumar, Angelique Kyriazis, Beatrice Alexandra Laitly, Joshua Markovski, Len Martin, Geoffrey McDonnell, Cindy Pan, Ruby Paroissien, Polly Reeves-Perrin, Michi Sano, Sebastian M. Schwarz, Alena Sipka, Michael Sullings, Jing Wei Yeong, William K. Cornwell

**Affiliations:** 1 School of Biological, Earth and Environmental Sciences, UNSW Sydney, Sydney, NSW, Australia; 2 Estate Management, UNSW Sydney, Sydney, NSW, Australia; 3 Sydney Arbor Trees, Botany, NSW, Australia; Tennessee State University, UNITED STATES

## Abstract

Large urban trees have many benefits. However, falling branches pose a serious hazard to both people and infrastructure. In several tree species, aerial roots grow down from branches to the ground. These roots are capable of thickening to support the branches, lessening the risk of tree failure. Unfortunately, in urban environments most aerial roots die before reaching the ground. Here, we report a new method for encouraging aerial roots to reach the ground, developed by the second-year botany class at UNSW Sydney. Our class tested three experimental treatments on aerial roots of *Ficus rubiginosa* Desf. ex Vent. (Port Jackson Fig)—PVC pipes filled with sphagnum moss, PVC pipes filled with potting mix, and PVC pipes filled with sphagnum moss and topped with funnels to catch extra rainwater. All three treatments significantly improved aerial root growth, with 26 of the 30 (87%) treatment roots reaching the ground after one year compared to 0 of the 10 control roots. Our method was successful for roots up to 3 m above the ground, suggesting the potential growth rate of aerial roots is substantial when conditions are favourable. Our novel approach is an attractive and cost-effective alternative to slings and other artificial supports. This project is an example of using undergraduate practical classes to teach science while simultaneously addressing important real-world problems.

## Introduction

Falling tree branches are a serious hazard, particularly in heavily populated areas such as city streets, university campuses and schools [1]. Tree failures cause substantial damage to property worldwide, and falling branches are responsible for many deaths each year. For example, 11% of all outdoor education-related deaths in Australia are caused by falling trees or tree branches [2]. Deaths from tree failures are particularly common during tropical cyclones and high winds [3, 4]. As extreme climate events like cyclones are predicted to increase in frequency and intensity [5], the damage caused by falling branches is also likely to rise in the coming decades. The problems caused by falling trees are not restricted to Australia, but apply in urban environments around the world.

Aerial roots can provide support to heavy branches in several plant taxa [6], including some members of the genera *Ficus* [7], *Metrosideros* [8], *Schefflera* [9], *Pandanus* [10], *Philodendron* [11] and *Anthurium* [12]. Many species in these genera are common as urban trees, both in the tropics and at higher latitudes. Aerial roots that grow from lateral branches to the ground form support columns that may help to stabilise and support trees and their branches, especially during storms and strong winds [13, 14]. However, aerial roots can grow slowly and they do not always do not always reach the ground [15]. Aerial root growth failure appears to be very common in urban environments worldwide. Worryingly, the extra weight of dangling aerial roots might actually increase the risk of tree or branch failure.

Despite the benefits of branches being supported by aerial roots, there is very little published literature on how to encourage and accelerate aerial root growth. There are well-established techniques in parts of India whereby *Ficus elastica* aerial roots are trained around bamboo or wood to form spectacular living bridges that can span more than 50m [16]. However, it is not clear whether winding roots around support structures accelerates root growth, or just trains the roots in a particular direction. Further, the challenges faced by aerial roots in an urban setting (where trees are often grown in isolation, with their aerial roots exposed to direct sunlight and drying winds) are likely rather different to the challenges faced by roots in moist broadleaf forests.

The main aim of our experiment was to determine whether root elongation in *Ficus rubiginosa* could be encouraged by providing more amenable growing conditions to existing aerial root clumps. Root elongation can be highly plastic in response to soil conditions [17], so we predicted that aerial roots surrounded by potting mix would elongate further in a year than control roots that hung in the air. There is also some evidence that aerial root growth can be inhibited by a lack of moisture. For example, moisture retention is a limiting factor to root growth and strength in vanilla [18], and the aerial roots of the tropical aroid *Anthurium clavigerum* grow twice as fast during the rainy season than in the dry season *[12].* This led us to predict that surrounding the roots by *Sphagnum* moss would result in greater aerial root elongation than achieved by roots in the air (control) or surrounded by potting mix. Mosses have previously been found to be helpful in retaining moisture in horticulture applications [19]. Finally, we predicted that providing a funnel to increase rainfall interception would further benefit aerial root growth.

This experiment was done in an unusual context. The project was initiated when Estate Management at UNSW Sydney approached professors of botany (WKC and ATM) for advice on how to encourage the growth of existing aerial root clumps towards the ground in the row of fig trees in the heritage-conservation area at Fig Tree Lane. These *Ficus rubiginosa* Desf. ex Vent. (Port Jackson Fig) trees were transplanted between 1893 and 1896 when the site was part of Kensington Racecourse. The fig trees and are now an iconic feature of the university. They are of environmental significance and arboreal heritage and are listed in the significant tree register for the council [20], in which they are described as “outstanding elements within this historic and culturally significant landscape precinct” [page 137, 20]. The trees are also of high ecological importance as they provide substantial habitat and resources to an array of fauna, as well as dramatically reducing the urban heat island effect [21]. Although each tree had multiple clusters of aerial roots on large branches, only a few roots very close to the trunks had grown to reach the ground. As such, support for these impressively large trees was almost exclusively contained in the subterranean and buttress roots, in addition to slings and wires that were installed in attempts to prevent branch failure.

The botany professors, together with Estate Management, decided to address the question of how to encourage the growth of aerial roots as a practical exercise with students in the Flowering Plants (BIOS2051) course at UNSW Sydney. The idea was to move away from standard undergraduate practical classes in which the answers are already known, and instead introduce the students to real-world science through an authentic project-based learning experience.

We present this work both as demonstration of a novel horticultural technique that can reduce the risks posed by large trees in an urban setting, and as a demonstration of the quality of science that can be achieved with an undergraduate science class.

Our hypotheses were:

1. That surrounding the roots by tubes with *Sphagnum* moss or potting mix would result in greater aerial root elongation than achieved by roots in the air (control).
2. That surrounding the roots with *Sphagnum* moss would result in greater aerial root elongation than achieved by roots surrounded by potting mix.
3. That providing a funnel to increase rainfall interception would result in greater aerial root growth than achieved in tubes without funnels.

## Methods

Aerial roots of all four individuals of *Ficus rubiginosa* located at Fig Tree Lane, UNSW, Sydney (33°54’ 59” S, 151°13’ 53” E) were selected for this study. This species sends down aerial roots in clusters which can form new trunks (also referred to as columns or props) when they make contact with the ground [22]. The fifth individual in this same line of trees was not included in this study as it was identified as *Ficus macrophylla* Desf. ex Pers. f. *macrophylla* (Moreton Bay Fig), which does not form new trunks from aerial roots. The trees are 16–30 metres in height, with canopies 18–35 metres wide. While the trees were originally planted in a much more open environment, the current environment around the trees is typical of a densely developed university setting, including tall buildings, walkways, and roads. Typical of highly urban trees, the trees’ crowns spread over a mix of low plantings, as well as asphalt and concrete.

Ten experimental blocks of four comparable adjacent roots that hung vertically over soil (rather than adjacent impenetrable surfaces) were selected for study. Two trees had one experimental block each, one tree had three experimental blocks, and one tree had five experimental blocks. Each experimental block contained one of each of the four treatments, assigned and arranged using a randomized block design. The four treatments consisted of (1) a PVC pipe filled with potting mix, (2) a PVC pipe filled with sphagnum moss, (3) a PVC pipe filled with sphagnum moss with a funnel attached at the top, and (4) a control, in which nothing was given to supplement the roots and they were left to grow normally. There was no pipe-only control, as without a growth media roots would simply blow out of pipes on windy days. The potting mix used contained 33% Australian Native Landscape supply of “Organic Garden Mix” (which is 50% black soil, 20% coarse sand, and 30% organics containing composted sawdust and organics and spent coffee grounds), 33% washed river sand, and 33% Cocopeat. To each soil bin of this mixture, we added 200ml of a general fertilizer (from a bulk mix containing: 250gm superphosphate, 500gm dolomite, 100gm trace elements, 200 gm blood and bone, 200gm ammonium nitrate, 200rm gypsum and 200gm lime) and 200gm of low Osmocote N16:P1.3:K9 slow release fertilizer. No additional water was applied to any of the treatment roots.

Experimental treatments were installed on 11th of October 2017. Immediately prior to installation, we measured the distance from the root tip to the ground. We also measured the horizontal distance between the top of each root and the trunk in order to determine if the distance from the root to parent trunk affected growth.

The PVC pipes were 9 cm in diameter and were cut in half lengthwise before installation to facilitate removal at the end of the experiment. Pipes were cut to match the length of the distance between root tip to the ground, plus an additional 50 cm (to prevent the roots being dragged out of the pipe during tree sway or movement due to wind), so varied in length from 150 cm to 400 cm. The two halves of the pipe were secured with zip ties, then tied to long wooden stakes that were firmly hammered in to the ground. The pipes were then filled to the top with the relevant medium (potting mix or sphagnum), with the roots inside. Funnels were made from aluminium sheet and measured ~30cm in diameter at the top and ~7cm diameter hole in the base. They were initially placed loosely on top of pipes however they were later secured using bolts due to fear of root damage caused by the metal funnel swaying in the wind. An elevated work platform was necessary to install and remove the treatments. We checked the treatments about half-way through the year, intending to top up the potting mix and sphagnum. However, all pipes remained filled to within a few cm of the top, so no maintenance was required.

**Fig 1:**
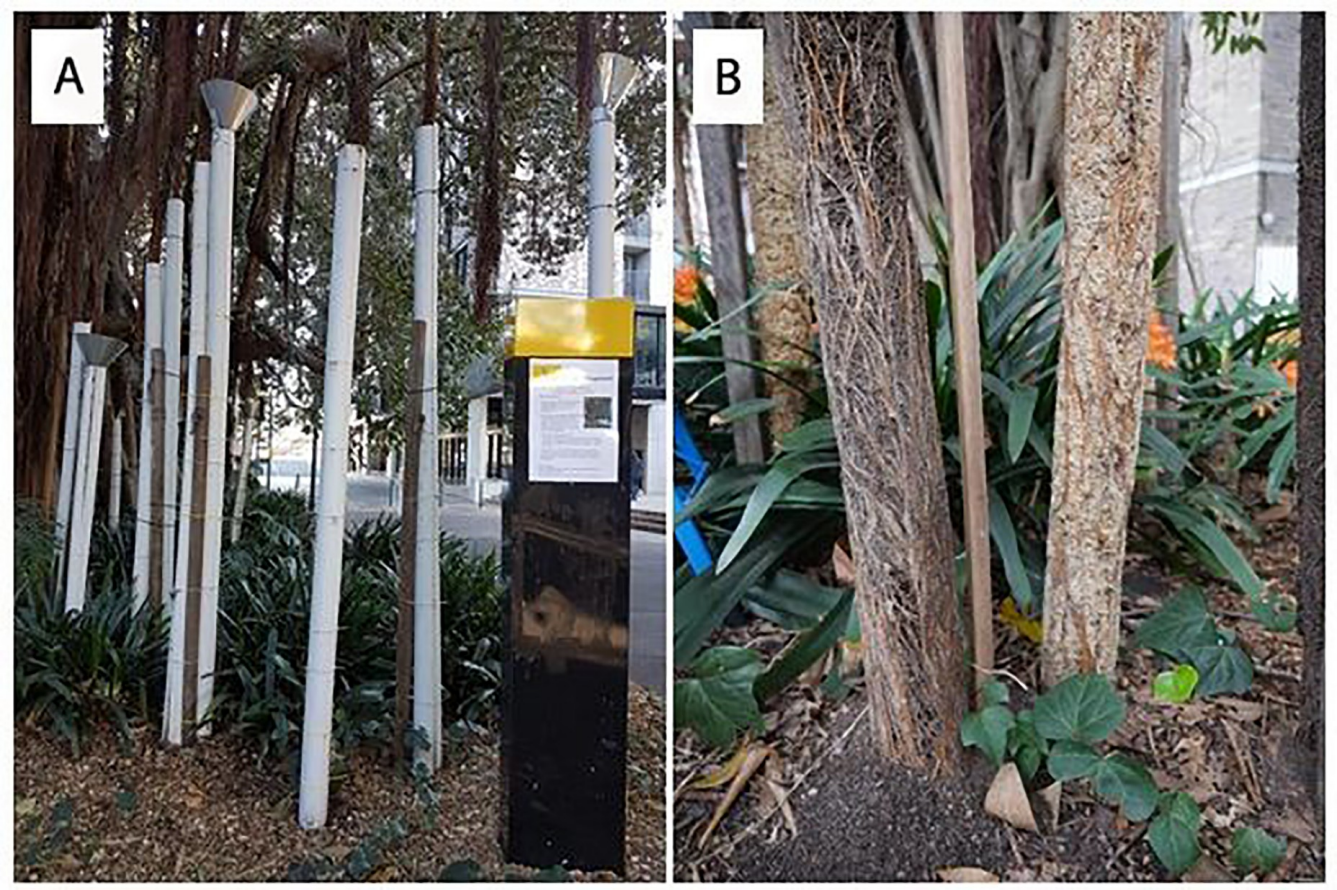
On campus fig tree root experiment provided important results and a strong project-based student learning experience. (a) The experimental treatments installed, showing the PVC tubes (some with funnels), and the informative sign outlining the project as these trees are in an area of high foot traffic. (b) Roots treated with potting mix (left) and sphagnum (right) had grown to fill the PVC tubes and extended firmly into the soil.

On the 17th of October 2018, we removed the treatment tubes. We then determined whether the aerial roots in each of the treatments had reached the ground (see Fig 1). If the roots had not reached the ground, the height of the root above the ground was measured. Data are provided in S1 Table and S2 Table.

On the 28^th^ of October 2019, we re-surveyed the roots, recording whether each root was grounded, and the diameter of the thickest root in each cluster was measured 10cm above the ground.

We analysed the proportion of roots reaching the ground with a generalized linear mixed model with treatment as a fixed effect, block as a random effect, and binomial errors [function glmer within the R library lme4 v1.1–17, 23]. To explore this in more detail we used the chi-square post-hoc tests [function chisq.post.hoc in the fifer v1.1 R library, 24]. We analysed average root growth rate (calculated as distance grown per unit time, following [25]) with a linear mixed effects model with treatment as a fixed effect and block as a random effect (lme4 v1.1–17, Bates et al. 2015). All analyses were done in R version 3.5.1.

## Results

**Fig 2:**
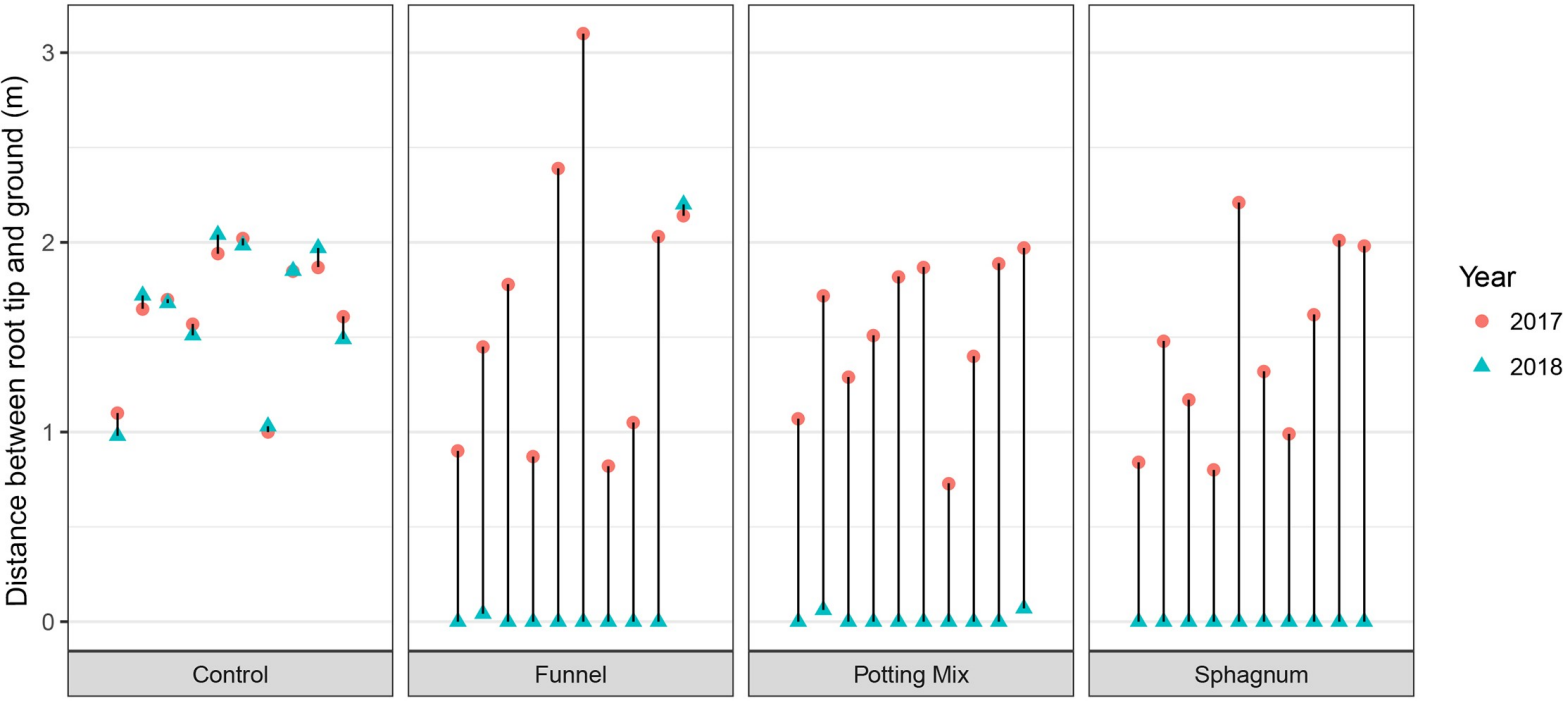
Change in distance between aerial root tip and ground over 1 year. Distance between aerial root tips and the ground (m) at the start (2017 –red) and the end (2018 –blue) of the experiment for four treatments. Each pair of points represents an individual root cluster. Roots are plotted in the order of which randomized block they were in.

Eight of the ten roots in the potting mix treatment, all of the roots in the sphagnum treatment and eight of the ten roots in the sphagnum with funnel treatment reached the ground. However, none of the ten control roots reached the ground (Fig 2). Many of the roots to which we applied treatments grew more than 2m within a year, and one grew more than 3m.

Including treatment as a fixed effect significantly improved the mixed model for both proportion of roots reaching the ground (P < 0.001) and average length of growth in the year (P < 0.001). All treatments were significantly better than the controls in both the probability of roots reaching the ground (all P < 0.01) and the average length grown in a year (all P < 0.001). There was no significant difference between the three treatment groups in either proportion of roots reaching the ground (all P > 0.05), or in the average length grown in a year (all P > 0.05; see results S1 Table in Supporting information).

The distance between the aerial root and the main trunk had no significant effect on root growth (P = 0.2). That is, our treatments are equally effective on distal roots as on those close to the parent trunk, which bodes well for management given that grounded aerial roots may be more valuable as stabilizing support structures when further away from the trunk.

**Fig 3:**
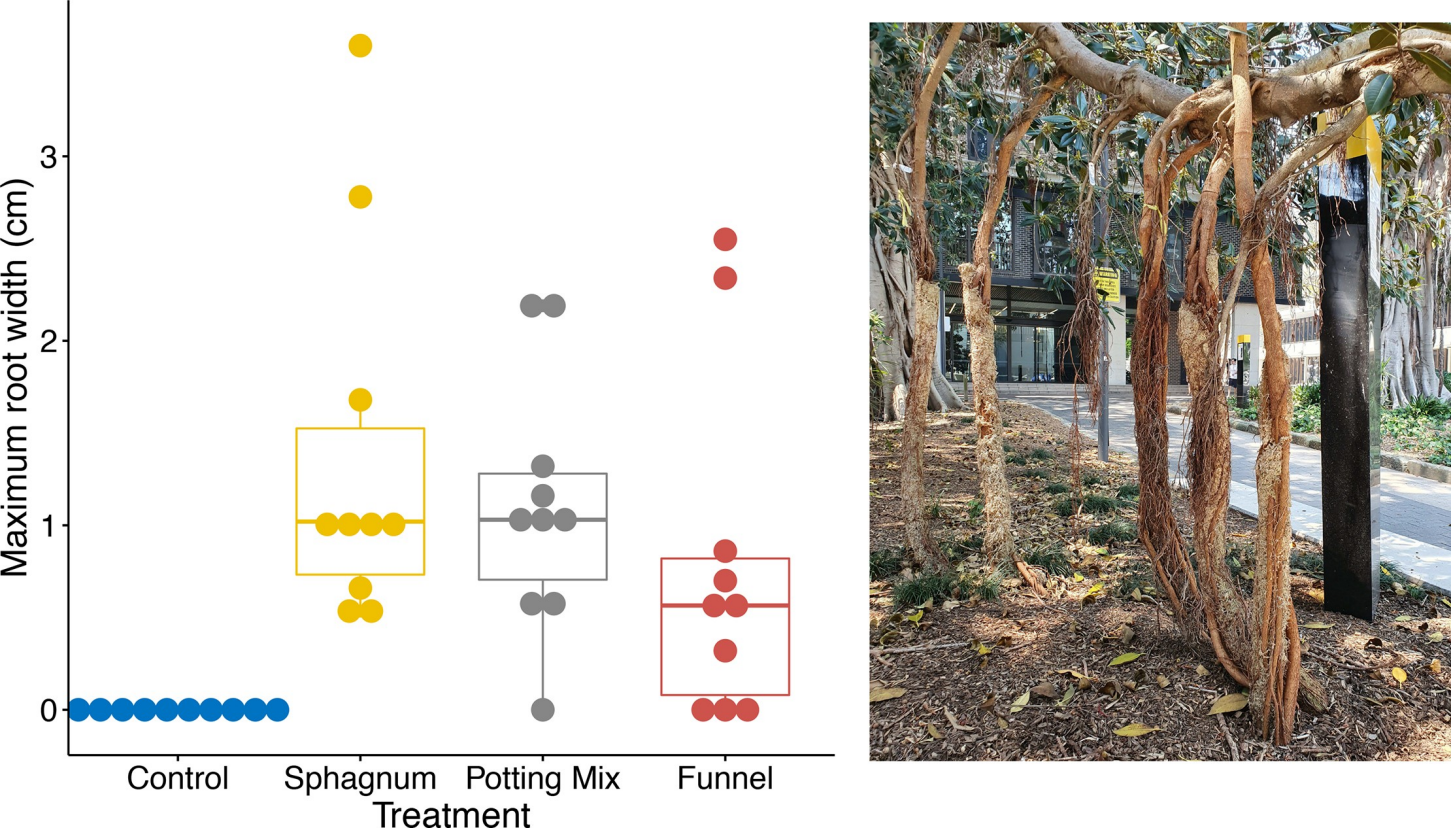
Growth of the aerial roots by two years after treatments were applied. Box plots showing the diameter of the thickest root (measured 10 cm above the ground on the 28^th^ of October 2019) in each of the four treatments, and a photograph showing the substantial nature of the root clusters. The curve visible in some of the roots resulted from the branch slumping when the supporting pipes were first removed in October 2018.

By October 2019 (24 months after the treatments were first applied), two of the previously anchored roots from the funnel treatment had snapped, but a further two roots had reached the ground (one from the potting mix treatment, one from the funnel treatment, both of which were within 7cm of the ground in October 2018). None of the control roots had reached the ground, so roots in the control treatment were still significantly (P < 0.01) less likely to reach the ground than were roots in the other three treatments (which were not significantly different from each other, P≥ 0.3). There were no significant differences in maximum root width (P > 0.15; Fig 3) between the funnel, potting mix and sphagnum treatments by October 2019. Six of the treatment roots had grown to more than 2cm in diameter, and they appear to provide structural support to the branches (Fig 3).

## Discussion

We devised and tested a method to facilitate the growth of aerial roots. Our treatments resulted in most of the treatment branches becoming firmly anchored in the ground within the course of a year (see Fig 1B), and developing into substantial support structures by the end of the second year (Fig 3). Our method does not require expensive materials, is relatively easy to install, and requires little to no maintenance. This method will allow arborists to strategically guide aerial-rooting trees to build their own living structural support, and decrease the risk of falling branches and tree failures. Our method also allows arborists to guide roots to the ground in the most desirable/practical locations, which is likely to be important for tree management in urban areas where roads, parking, and clear walkways are high priorities. We hope that applying this method will allow for greater retention of large urban trees, as these are known to have numerous benefits including air pollution reduction [26], carbon sequestration [27], enhanced human happiness [28], and increased property values [29]. In particular, we hope that this technique can be used to enhance the longevity of large urban trees both in Australia and elsewhere in the world.

There was no significant difference in the efficacy of the sphagnum, potting mix and sphagnum with funnel treatments in our study. The funnels add expense and complexity, but do not enhance growth. We included the funnel treatment because we hypothesized that the extra rain catchment area might enhance root growth. However, a substantial amount of water flows down branches and trunks during rainfall events [30]. That is, the lack of an effect of funnels might be because the majority of the water may have reached the tubes through stem flow rather than as free-falling precipitation. Similarly, sphagnum is relatively expensive and did not lead to better results than simple potting mix. Sphagnum was more difficult to pack into the tubes than potting mix, and air pockets in the sphagnum led to patches with noticeably weaker root growth (*pers*. *obs*. ATM). Additionally, sphagnum is often unsustainably harvested [31]. We therefore recommend the use of tubes without funnels, filled with potting mix for future application of this method. We would also recommend that pipes are left on until the roots form strong columns into the soil.

When we removed the treatment tubes, we noticed a relatively high abundance of ants and other invertebrates making use of the protected habitat on and around the roots. Some of these invertebrates were likely herbivores. Thus, occasional insecticide treatment might be beneficial if this technique is applied to a species that is highly susceptible to root herbivory, or in individuals or environments with high pest abundance.

In control treatments in which roots were left to grow on their own, we recorded a 0% success rate for getting aerial roots to the ground. We do not know the mechanism that causes the failure of aerial roots in urban environments. However, the success of the manipulations suggests that whatever the disadvantageous aspect of the urban environment was, it was ameliorated by all of our treatments. Aerial roots are known to be very successful at reaching the ground in high humidity, low wind, low vapour pressure deficit environments such as those found in the understory of closed canopy forests [32]. In contrast, compared to trees in closed canopy forests, trees in urban environments likely experience warmer temperatures and a lower leaf area index environment, as well as more under-canopy breeze and periodic high wind and high temperature events [33, 34]. Such a harsh climate could result in aerial root desiccation and be unsuitable for their growth in *Ficus rubiginosa* [22]. The potting mix or *Sphagnum* could have then increased the boundary layer around the root tips and decreased the potential for desiccation. If it is in fact lack of a suitably moist environment that prevents aerial roots in urban settings (such as those in our control group) from growing, then the rates of root growth that we recorded are even more remarkable given that our experiment ran during a period of relatively low rainfall (773 mm during our experiment, compared to mean annual precipitation of 1083 mm; data from Sydney Airport weather station, Bureau of Meteorology).

Because the aerial roots are now likely to be part of the structural support for the branches (Fig 3), we were not able to cut the aerial roots to study their anatomy (weakening the trees in the urban setting might be dangerous, and we cannot damage these heritage listed trees). However, an interesting direction for future study would be to determine whether the anatomical structure of the aerial roots changed as a result of growing in the substrate within the tubes. Aside from the intellectual interest of this question, it is important because the amount of structural support that the roots provide will depend on the anatomy of the roots. There are already hints that the aerial roots of *Ficus* can have substantial mechanical strength–for example, the aerial roots of *Ficus elastica* can be trained to form living bridges that span more than 50m [16].

Undergraduate students were involved from the initial establishment of our experiment through data collection, analysis, figure preparation and writing. Thus, in addition to its primary scientific aim, this project gave an undergraduate science class invaluable experience in scientific research. In too many science practical classes, students run through the same exercises year after year, leaving a limited sense of discovery and giving a false impression of how straightforward the scientific process is. Although we do not have quantitative data on the efficacy of this project as a teaching exercise, involving students in real-world science gave a substantially better experience for both academics and students alike, in addition to the benefits to the university community (in developing a solution to a potentially dangerous issue on campus) and to the scientific community (in the publication of novel application focused research, and in the training of capable scientists).

In summary, we have developed a highly effective method for enhancing aerial root growth in *Ficus rubiginosa*. Our method is cheap and relatively easy to apply, and the establishment of supportive aerial roots are more aesthetically pleasing than other commonly used methods for preventing failure of heavy branches, such as slings and artificial support structures. While we have tested this technique on only one tree species, it seems likely that a similar approach should be effective in other species which put down aerial roots. We hope that our findings will be applied in urban environments to reduce injuries or damage caused by tree failures, and to allow private land-owners, universities, botanic gardens and councils to retain their majestic old trees.

## Supporting information

S1 Table Posthoc pairwise comparisons of three treatments and a control in 2018. There were no significant differences between the treatments in either proportion of roots reaching the ground by October 2018, or in average length of root growth in a year (2017–2018). However, all treatments were significantly greater than the controls in both of these measures. Detail of analyses are provided in methods.
(DOCX)

S2 Table Posthoc pairwise comparisons of three treatments and a control in 2019. There were no significant differences between the treatments in either proportion of roots anchored to the ground by October 2019, or in the diameter of the thickest root in October 2019 (diameter measured 10cm above the ground). However, all treatments were significantly greater than the controls in both of these measures. Detail of analyses are provided in methods.
(DOCX)

S1 Datasheet Fig aerial root project datasheet. (CSV)
