# Supplementary material for: From dangerous branches to urban banyan: Facilitating aerial root growth of Ficus rubiginosa
Source: PLoS One. 2019 Dec 30;14(12):e0226845. doi: 10.1371/journal.pone.0226845 (PMC6936823; doi:10.1371/journal.pone.0226845)
Supplement: S1 Table — There were no significant differences between the treatments in either proportion of roots reaching the ground by October 2018, or in average length of root growth in a year (2017–2018). However, all treatments were significantly greater than the controls in both of these measures. Detail of analyses are provided in methods. (DOCX) [file pone.0226845.s001.docx]

**Supporting information**

**Moles et al. From dangerous branches to urban banyan: Facilitating aerial root growth of *Ficus rubiginosa***

**Table S1:** Posthoc pairwise comparisons of three treatments and a control in 2018. There were no significant differences between the treatments in either proportion of roots reaching the ground by October 2018, or in average length of root growth in a year (2017-2018). However, all treatments were significantly greater than the controls in both of these measures. Detail of analyses are provided in methods.

|  | ***Average length grown in a year*** | ***Proportion of aerial root tips meeting the ground*** |
| --- | --- | --- |
| **Treatment pair** | **P value** | **P value** |
| Control – Funnel | <0.001 | 0.0014 |
| Potting mix – Funnel | 0.983 | 1.000 |
| Sphagnum – Funnel | 1.00 | 0.5684 |
| Potting mix – Control | <0.001 | 0.0014 |
| Sphagnum – Control | <0.001 | <0.001 |
| Sphagnum – Potting mix | 0.989 | 0.5684 |
