# Supplementary material for: From dangerous branches to urban banyan: Facilitating aerial root growth of Ficus rubiginosa
Source: PLoS One. 2019 Dec 30;14(12):e0226845. doi: 10.1371/journal.pone.0226845 (PMC6936823; doi:10.1371/journal.pone.0226845)
Supplement: S2 Table — There were no significant differences between the treatments in either proportion of roots anchored to the ground by October 2019, or in the diameter of the thickest root in October 2019 (diameter measured 10cm above the ground). However, all treatments were significantly greater than the controls in both of these measures. Detail of analyses are provided in methods. (DOCX) [file pone.0226845.s002.docx]

**Table S2:** Posthoc pairwise comparisons of three treatments and a control in 2019. There were no significant differences between the treatments in either proportion of roots anchored to the ground by October 2019, or in the diameter of the thickest root in October 2019 (diameter measured 10cm above the ground). However, all treatments were significantly greater than the controls in both of these measures. Detail of analyses are provided in methods.

|  | ***Diameter of thickest root*** | ***Proportion of aerial root tips meeting the ground*** |
| --- | --- | --- |
| **Treatment pair** | **P value** | **P value** |
| Control – Funnel | 0.03 | 0.006 |
| Potting mix – Funnel | 0.65 | 0.70 |
| Sphagnum – Funnel | 0.15 | 0.32 |
| Potting mix – Control | 0.002 | 0.0004 |
| Sphagnum – Control | 0.001 | 0.0001 |
| Sphagnum – Potting mix | 0.73 | 1.0 |
